# A comprehensive survey of integron-associated genes present in metagenomes

**DOI:** 10.1186/s12864-020-06830-5

**Published:** 2020-07-20

**Authors:** Mariana Buongermino Pereira, Tobias Österlund, K Martin Eriksson, Thomas Backhaus, Marina Axelson-Fisk, Erik Kristiansson

**Affiliations:** 1 Department of Mathematical Sciences, Chalmers University of Technology, Gothenburg, Sweden; 2 Centre for Antibiotic Resistance Research (CARe) at University of Gothenburg, Gothenburg, Sweden; 3 Department of Biological and Environmental Sciences, University of Gothenburg, Gothenburg, Sweden; 4 Gothenburg Centre for Sustainable Development, Chalmers University of Technology, Gothenburg, Sweden

**Keywords:** Integrons, Metagenomics, Gene cassettes, Functional annotation, ORFans, Antibiotic resistance, Horizontal gene transfer

## Abstract

**Background:**

Integrons are genomic elements that mediate horizontal gene transfer by inserting and removing genetic material using site-specific recombination. Integrons are commonly found in bacterial genomes, where they maintain a large and diverse set of genes that plays an important role in adaptation and evolution. Previous studies have started to characterize the wide range of biological functions present in integrons. However, the efforts have so far mainly been limited to genomes from cultivable bacteria and amplicons generated by PCR, thus targeting only a small part of the total integron diversity. Metagenomic data, generated by direct sequencing of environmental and clinical samples, provides a more holistic and unbiased analysis of integron-associated genes. However, the fragmented nature of metagenomic data has previously made such analysis highly challenging.

**Results:**

Here, we present a systematic survey of integron-associated genes in metagenomic data. The analysis was based on a newly developed computational method where integron-associated genes were identified by detecting their associated recombination sites. By processing contiguous sequences assembled from more than 10 terabases of metagenomic data, we were able to identify 13,397 unique integron-associated genes. Metagenomes from marine microbial communities had the highest occurrence of integron-associated genes with levels more than 100-fold higher than in the human microbiome. The identified genes had a large functional diversity spanning over several functional classes. Genes associated with defense mechanisms and mobility facilitators were most overrepresented and more than five times as common in integrons compared to other bacterial genes. As many as two thirds of the genes were found to encode proteins of unknown function. Less than 1% of the genes were associated with antibiotic resistance, of which several were novel, previously undescribed, resistance gene variants.

**Conclusions:**

Our results highlight the large functional diversity maintained by integrons present in unculturable bacteria and significantly expands the number of described integron-associated genes.

## Background

Integrons are machineries that enables transfer of genetic material between DNA molecules [1, 2]. Through site-specific recombination, integrons have the ability to incise, excise and re-organize genes into, out of, and within a host genome [3–5]. Integrons are estimated to be present in at least 6% of the bacterial genomes [6] and can be located either on chromosomes, as in e.g. *Vibrio ssp*. and *Xanthomonas ssp.*, or on conjugative elements, as is common for in pathogens such as *Escherichia coli* and *Salmonella enterica* [7, 8]. Since integrons enable incorporation of a wide range of genes, they have been suggested to play a major role in the adaptation and evolution of many forms of bacteria [9–11]. Integrons present in pathogenic bacteria often carry antibiotic resistance genes, which enable the bacteria to survive antibiotic treatment. Similarly, chromosomal integrons present on *Vibrio ssp.* maintain virulence factors, such as genes encoding for toxins, which enable bacteria to gain advantages when colonizing different environments and hosts [7, 12, 13]. However, despite their central role in adaptation, the functional repertoire of integron-associated genes is far from fully characterized.

All integrons are organized according to a common structure. First, they carry an *intI* gene which encodes an integrase, the enzyme that facilitates the gene transfer by sequential incorporation of genes at the *attI* recombination site. Furthermore, there is an integron-associated promoter (Pc) that regulates the expression of the incorporated genes. Genes mediated by the integron are organized in gene cassettes. Each cassette consists of an open reading frame (ORF) together with an *attC* recombination site [9, 14]. *AttC* sites are imperfect palindromic sequences that are 55 to 141 nucleotides long and exhibit a very low degree of conservation between gene cassettes [4, 15]. During the gene transfer, the bottom strand of the *attC* site folds into a hairpin secondary structure through alignment of two pairs of complementary motifs, R”/R’ and L”/L’ that are separated by short spacers, which are up to 10 nucleotides long. The L-sites are separated by a region that is 14 to 102 nucleotides long and forms the central loop of the hairpin. R’ and R” are the most conserved parts of the *attC* site and have the general motifs RYYYAAC and GTTRRRY, respectively (where R is a purine and Y is a pyrimidine). Integrons located on conjugative elements usually consist of up to 8 gene cassettes, many of them with antibiotic resistance genes, while chromosomal integrons can carry hundreds of gene cassettes, which can be spread over the chromosome in multiple arrays [7].

Multiple efforts have been made to study integron-associated genes and their biological functions. The integron database INTEGRALL contains, for example, roughly 1500 integrase and 8000 gene cassettes extracted from public sequence repositories [16]. Also, in a recent study, 2,484 genomes from bacterial isolates were analyzed for the presence of integrons which resulted in 4,597 predicted *attC* sites [6]. Most bacteria are, however, hard to cultivate under standard lab conditions and their genome is therefore not yet sequenced [17, 18]. Analysis based on genomes from bacterial isolates will thus reflect only a small proportion of the integron-associated genes. To this end, metagenomics offers a cultivation-independent way to analyze the genetic basis of bacterial communities. Indeed, studies using targeted amplicon sequencing have shown that integrons are common in bacterial communities in the environment and the human microbiome [19–24]. However, amplicon-based studies have so far mainly targeted specific types of integron classes or structures (often integrases of class I) and they are therefore unable to capture the full diversity of integron-associated genes. Shotgun metagenomics is, in contrast, free from many of the biases associated with amplicon sequencing and can thus describe the functional potential of a bacterial community in a more holistic way, including the genes located in integrons. However, metagenomic sequence data is fragmented and needs to be assembled prior analysis - a process that is often especially hard for integrons due to their repetitive nature [23, 25]. Consequently, complete fully reconstructed integrons are rare in metagenomic data, which makes their identification and the study of their incorporated gene cassettes challenging.

In this study, we present a comprehensive survey of integron-associated genes present in metagenomes. We used a novel computational approach optimized for highly fragmented sequence data, where the individual *attC* sites were first detected and then, in a second step, their associated upstream ORFs were identified. This circumvented the need for assembled full-length integrons. We analyzed 375 million contigs assembled from approximately 10 terabases of raw metagenomic data and found 13,397 non-redundant integron-associated genes. The highest abundance of integron-associated genes was found in marine environments, where they were approximately a 100-fold more common than in the human microbiome. The identified genes encoded proteins with a large functional diversity. The most abundant functional classes included defense mechanisms and gene mobility which were also highly overrepresented among the integron-associated genes. We noted furthermore, that genes associated with toxin-antitoxin systems as well as glutathione s-transferases (GST) were especially common. Interestingly, as many as two-thirds of the integron-associated genes had an unknown function and could not be matched to any database. Moreover, less than 1% of the integron-associated genes were antibiotics and biocide/metal resistance genes of which several were novel variants that had not been previously described. In addition, our results describe the extensive functional repertoire associated with bacterial integrons and significantly expand the number of known integron-associated genes.

## Results

**Fig. 1:**
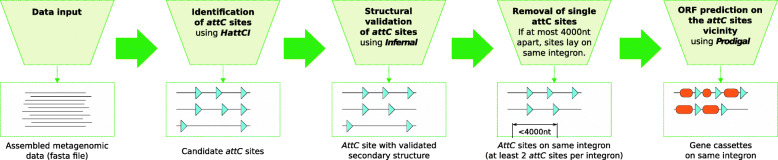
Description of the computational pipeline used to detect *attC* sites in metagenomic data. Assembled metagenomic DNA sequences are used as input. Next, the gHMM-based HattCI is used to detect the *attC* sites present in the input sequences. Subsequently, the secondary structure of the detected *attC* sites is evaluated by a covariance model implemented in Infernal, which runs the search in its most sensitive mode. Identified *attC* sites on the same strand are considered to be part of the same integron when they are at maximum 4,000 nucleotides (nt) apart. Note that integrons with only one *attC* site are removed from the analysis in order to ensure a high true positive rate. Finally, the ORFs are predicted upstream of the *attC* sites

Assembled metagenomic data was analyzed for integron-associated genes using a newly developed computational pipeline (Fig. 1). First, putative *attC* sites were identified based on their evolutionarily conserved patters using HattCI [15], which implements a generalized Hidden Markov model (gHMM) that individually describes each motif present in the *attC* site (R’, R”, L’, L”, spacers and loop). Next, the secondary structures of the identified *attC* sites were validated using a covariance model implemented using Infernal [26]. The model was trained on a structure-based multiple alignment of previously identified and manually annotated *attC* sites. Afterwards, the results were filtered to remove potential false positives, for that we excluded predicted *attC* sites that were isolated on the sequence and thus not located in close vicinity to any other *attC* site (maximum distance between *attC* sites was set to be 4,000 nucleotides, which was chosen as a conservative upper limit for the gene length in the cassettes). Finally, Prodigal [27] was used to predict open reading frames (ORFs) upstream of the *attC* sites for the top strand. Evaluation based on 291 gene cassettes demonstrated that the pipeline had a sensitivity of 91% for detecting *attC* sites. The false positive rate was low with not a single incorrect match in 400 gigabases of sequence data generated by reshuffling eight bacterial genomes. See Methods for full details about the computational pipeline implementation and the evaluation.

The pipeline was used to analyze more than 10 terabases of metagenomic data assembled into 370 million contigs comprising 267 gigabases. The sequence data, which was collected from four major databases and ten metagenomic studies, reflected a wide range of different microbial communities (Table 1). Applying the pipeline to the full dataset resulted in 16,148 predicted gene cassettes, comprising 11,585 unique *attC* sites and 13,397 unique ORFs (Additional file 1: Table S1).

**Table 1:** Size of each dataset in terms of assembled gigabases and number of sequences, together with the number of predicted *attC* sites and ORFs

	**Gigabases of assembled data**	**Number of sequences**	**Number of predicted*attC*sites1**	**Number of predicted ORF’s**
**Dabases**				
CAMERA [73]	66	179,126,552	354 (0.005)	360
MG-RAST [74]	13	7,881,749	5,377 (0.4)	6,471
NTenv (GenBank) [75]	87	86,661,686	5,094 (0.06)	6,467
EBI Metagenomics [76]	3	3,886,782	1,283 (0.4)	1,668
**Other Datasets**				
Tara Oceans [81]	61	57,540,959	2,746 (0.05)	3,507
Aquatic microbiome [82]	1	4,094,883	2 (0.002)	2
Marine biofilm^2^	3	2,046,453	1,440 (0.5)	1,909
Human gut [83]	10	6,589,348	2 (0.0002)	2
Human gut from diabetic patients [84]	2	891,652	2 (0.001)	2
Human gut from travelers [85]	18	20,555,914	14 (0.0008)	14
Elephant gut [86]	1	311,295	29 (0.03)	41
Corn and prairie crops soil [87]	2	4,944,181	29 (0.02)	30
Microbial fuel cells [88]	0.15	207,982	38 (0.3)	42
Subarctic microbiomes [89]	0.04	169,650	2 (0.05)	2
**Total**	**267**	**374,739,436**	**16,376**	**20,517**
			**(11,585**^**3**^**)**	**(13,397**^**4**^**)**

^1^In parenthesis, copies per million bases.

^2^Prepared by the authors.

^3^Non-redundant hits.

^4^Non-redundant hits. Aminoacid sequences

**Fig. 2:**
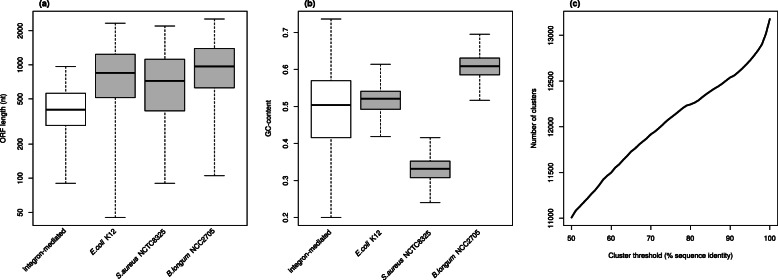
Boxplots for **a** ORF length and **b** G/C-content for the integron-associated genes identified in this study. For comparisons, the corresponding data for three reference bacterial species have been included, *Escherichia coli* K-12, *Staphylococcus aureus* NCTC8325 and *Bifidobacterium longum* NCC2705. **c** Cluster analysis of the integron-associated genes. The x-axis shows the cluster threshold in sequence identity (higher value corresponds to a more homogeneous clusters) and the y-axis the number of produced clusters

The relative abundance of *attC* sites varied between 0.0002 and 0.5 copies per million bases. The highest abundance was found in marine biofilm communities while the level was lowest in the human microbiome. A catalog of the predicted integron-associated genes was formed based on the set of unique ORFs. The length of the genes in the catalog was short, with a median of 402 nucleotides and a standard deviation of 308 nucleotides (Fig. 2a). This was close to the length of the previously identified integron-associated genes reported in the INTEGRALL database [16] (median 474, sd 290) but considerably shorter than the lengths of chromosomal bacterial genes (median 831, sd 735) (Fig. 2a). The G/C-content of the genes in the catalog varied substantially and was between 0.20 and 0.74 with a median of 0.50 and a standard deviation of 0.09. Similar to the gene length, the G/C-content corresponded well with the one found in the genes in INTEGRALL (median 0.51 and standard deviation 0.08). The G/C-distribution was however much wider than what is typically encountered within a single bacterial genome where the G/C-content standard deviation was between 0.04 and 0.05 (Fig. 2b).

Next, the diversity of the catalog was assessed using cluster analysis. At a 97% amino acid sequence similarity cut-off, the 13,397 genes formed 12,833 clusters (Fig. 2c), which decreased to 11,946 clusters at a 70% cut-off. At a 50% cut-off, there were still 11,007 clusters formed of which the largest contained 30 genes while 9,517 clusters were singletons. Thus, the number of clusters reduced slowly with a decreasing sequence similarity cut-off, indicating a high diversity with many distinct genes.

**Fig. 3:**
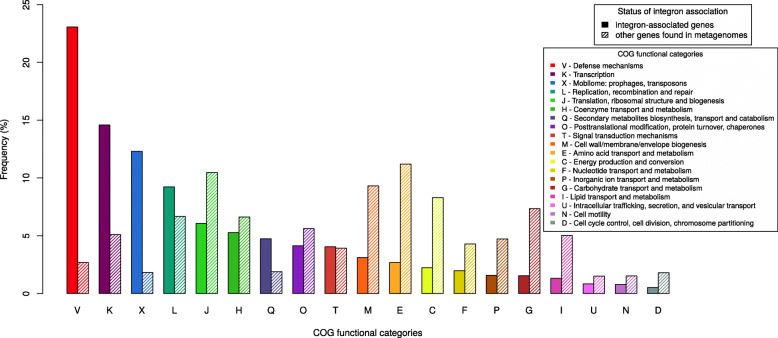
Functional annotation of the integron-associated genes (solid bars) and other genes found in metagenomes using COG functional categories (striped bars). Of the 13,397 integron-associated genes in our catalog, 2,277 genes matched a COG with a known function. 116,259,264 ORFs were not associated with integrons in metagenomes, out of which 50,201,496 matched a COG with a known function. Percentages on the plot are given in relation to those numbers

The gene catalog was functionally annotated by comparing the genes against three different databases containing functional profiles: Cluster of Orthologous Groups (COG) [28], TIGRFAM 15.0 [29] and PFAM 29.0 [30]. In total, 4817 (36%) of the genes had a match (E-value<10^−5^) against at least one of the three databases, where 3,497 (26%), 1,727 (13%) and 4,373 (33%) of the ORFs matched functions in the COG, TIGRFAM and PFAM databases, respectively. Among those were 2,277 (17%), 1,203 (9%) and 3,488 (26%) matched to profiles with a known biological function. The most highly abundant functions included toxin-antitoxin systems (e.g. TIGR02607, TIGR02385, PF05016, PF02604, COG2026), GST, in particular, glutathione-dependent formaldehyde-activating genes (PF04828, TIGR02820, COG3791) as well as acetyltransferases (TIGR01575, PF13302, COG0454), endonucleases (PF01844, PF14279), receptor-associated transport activity (TIGR01352) and methylases (COG0863) (Additional file 1: Table S1). The matches to the COG database were assigned to 24 major functional classes (‘COG categories’). The most common functional classes were defense mechanisms (23%) followed by transcription (15%) and mobility (12%). For the TIGRFAM database, the most common functional classes (‘TIGRroles’) were extra-chromosomal functions (29%), protein synthesis (11%) and DNA metabolism (10%) (Additional file 2: Fig. S1b). Gene ontology analysis, based on the matches to the PFAM databases showed that the most common molecular function found is associated with catalytic activities (1.3%), while the most common biological process is related to metabolism (1.1%) and the most common cellular component is part of the membrane (0.42%) (Fig. 3 and Additional file 3: Table S2)).

**Fig. 4:**
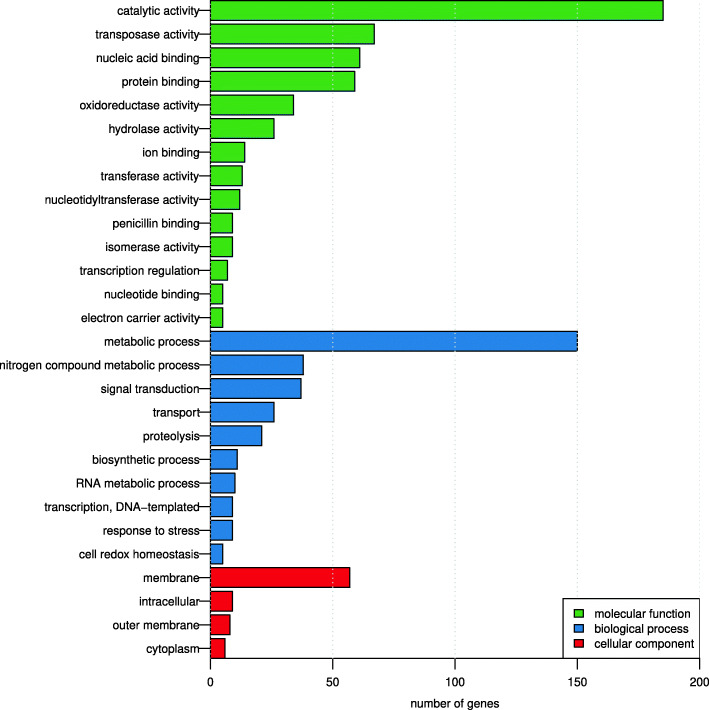
Gene ontology analysis of the integron-associated genes using PFAM families. Out of the 13,397 integron-associated genes in our catalog, 3,488 matched a PFAM family with a known function, which were in turn mapped to the metagenomics GO slim. Not all PFAM families mapped to a GO term; as a result, 1534 genes had a corresponding GO term. Level 1 terms were removed and those with at least 5 counts were kept (For the whole list GO terms and their counts please see Additional file 3: Table S2)

Next, we assessed which functional categories were most overrepresented among the integron-associated genes compared to other genes present in the metagenomic data (Fig. 4 and Additional file 4: Table S3)). Using Prodigal, we predicted 116,259,264 unique ORFs that were not associated with any *attC* site, of which 50,201,496 (43%) matched a COG with a known function. The difference in functional assignments between the two groups of genes was assessed for each COG category using Fisher’s exact test. The three COG categories that were most overrepresented among the integron-associated genes were defense mechanisms (odds ratio 6.46,*p*<10^−15^), mobilome (odds ratio 5.06,*p*<10^−15^) and function unknown (odds ratio 3.66,*p*<10^−15^). Categories that instead were most underrepresented among the integron-associated genes included carbohydrate metabolism and transport (odds ratio 0.158,*p*<10^−15^), amino acid transport and metabolism (odds ratio 0.180,*p*<10^−15^) and lipid transport and metabolism (odds ratio 0.197,*p*<10^−15^).

Next, the catalog was compared to functionally specialized databases containing integron-associated genes (INTEGRALL), antibiotic resistance genes (ResFinder) [31] and biocide and metal resistance genes (BacMet) [32] (Table 2). Interestingly, only 51 (0.38%) of the genes in the catalog had a close match (sequence similarity>97%) to genes previously reported in INTEGRALL. The majority of these genes were either previously known integron-associated resistance genes, hypothetical proteins or genes with unknown function. At a more relaxed sequence similarity cut-off (>70%), the overlap with INTEGRALL increased, but only to 201 (1.5%). The low number of matches to INTEGRALL suggests that the large fraction of the ORFs in the catalog is previously undescribed. The catalog also contained few known antibiotic, metal and biocide resistance genes. Only 25 (0.19%) and 4 (0.030%) of the genes had a close match to genes in the ResFinder and BacMet databases respectively. These matches included several previously reported integron-associated resistance genes, such as the *β*-lactamases VIM, OXA-2 and OXA-10, the sulfonamide resistance gene sul1, the aminoglycoside resistance genes aadA and the quaternary ammonium compound-resistance protein qacF (Additional file 1: Table S1). Interestingly, when the matching criterion was set to 70% sequence similarity, the number of matches increased to 31 (0.23%) and 7 (0.052%) for ResFinder and BacMet respectively, suggesting the presence of integron-associated resistance genes previously uncharacterized in the literature. Novel putative resistance gene variants included a class D *β*-lactamase with 93% similarity to OXA-9, several trimethoprim resistance genes ranging between 77% to 96% similarity to known dfr-genes and chloramphenicol resistance gene with 88% similarity to catB (Additional file 1: Table S1).

**Table 2:** Results from blast searches against the integron database INTEGRALL, and antibiotic and metal resistance databases, ResFinder and BacMet, respectively. Similarity thresholds used were 70% and 97%

**Database**	**> 70%**	**> 97%**
INTEGRALL [16]	201 (1.5%)	51 (0.38%)
ResFinder [31]	31 (0.23%)	25 (0.19%)
BacMet [32]	7 (0.052%)	4 (0.030%)
**Total (% of integron-associated genes)**	**239 (1.8%)**	**80 (0.60%)**

Finally, structure-based clustering was done to investigate the association between biological function and structure of the *attC* sites. Based on GraphClust [33], 4102 *attC* sites were clustered into five distinct groups containing 319 to 1928 *attC* sites each (Additional file 5: Fig. S2). The remaining 7483 *attC* sites were removed since GraphClust either 1) assigned them to a cluster with an invalid structural consensus or 2) could not assign them unambiguously to a specific cluster. Tests for overrepresentation showed that several groups were significantly associated with specific COG categories (Additional file 6: Table S4) and GO terms (Additional file 7: Table S5). In particular for the COG categories, clusters (a) and (c) were associated with defense mechanisms (*p*-values 0.019 and 0.00034, respectively), cluster (b) with inorganic ion transport and metabolism (*p*-value 0.0272), cluster (d) with cell wall/membrane/envelope biogenesis (*p*-value 0.0030) and cluster (e) with secondary metabolites biosynthesis, transport and catabolism (*p*-value 8.6x10^-5^).

## Discussion

In this study we applied a computational pipeline to metagenomic data and identified 13,397 integron-associated genes present in the environment. The analysis was based on 370 million contigs assembled from approximately 10 terabases of sequence data representing microbial communities from a wide range of environments, including the human microbiome. This is, to the best of our knowledge, the most comprehensive characterization of integron-associated genes in uncultured bacteria to date. Indeed, only a small proportion of the identified genes (51 out of 13,397) has previously been reported in the extensive INTEGRALL database, which suggests that most of our findings are not represented in public repositories. Analysis of the identified genes showed a high functional diversity, where only 36% of the genes could be assigned to a known biological function. The functional role of as many as 64% remained unknown. In addition, structured-based clustering of *attC* sites resulted five groups which showed a weak, but significant, association with specific biological functions.

The relative abundance of gene cassettes differed substantially between the analyzed metagenomes; the levels were found to be especially high in the epipelagic and mesopelagic communities and biofilms. Here, the number of *attC* sites ranged between 0.05 and 0.50 copies per million bases, which, assuming an average genome size of 2 megabases [34], corresponds to up to approximately 1 gene cassette per cell. High levels of horizontal mobile elements and, in particular, integrons, have previously been reported in marine microbial communities. For example, a large diversity of integrases as well as gene cassettes has been described in marine sediments [20, 35] and deep-sea hydrothermal vent fluid [19]. Also, integrase genes have previously been reported to be common in marine periphyton biofilms [36]. Many forms of bacterial species commonly occurring in marine ecosystems, such as *Vibrio spp.* [13] and *Pseudomonas spp.* [37], are known to maintain chromosomal integrons, which may contribute to the high level of gene cassettes observed in these environments [7, 38, 39]. In contrast, low levels of integron-associated genes were found in the human gut metagenomes. Indeed, we found less than 0.01 gene cassettes per cell, which is a 100-fold lower abundance than in the marine metagenomes. This suggests that integron-associated genes are relatively rare in the human microbiome. These findings are in line with previous studies where the abundance of integron-associated integrases has been shown to be substantially lower in the human microbiome compared to many other microbial communities [40]. It should, however, be pointed out that these results will, most likely, not reflect the true diversity of integron-associated genes in any of these environments. Microbial communities are highly diverse and, due to limited sequencing depth, metagenomic studies will only describe integron-associated genes with highest abundance. Nevertheless, our results underline that there are substantial differences in the abundance of integron-associated genes between environmental compartments.

Functional analysis of the 13,397 integron-associated genes demonstrated a large functional diversity and a wide range of biochemical roles. Commonly occurring functional classes included defense mechanisms, gene mobility, transcription, protein synthesis, DNA metabolism and gene expression regulation. Genes associated with defense mechanisms and mobility were highly overrepresented and more than five times more common among genes in integrons than among other genes in the communities. Moreover, toxin-antitoxin systems (TA-systems) were found to be especially common in the gene catalog. TA-systems typically contains two types of genes, one that encodes a toxin that can destroy the bacterial cell and one that encodes an antitoxin that inhibits the toxin. The eventual loss of the antitoxin gene(s), caused by illegitimate recombination events that impairs genes in the integrons, would allow the toxin to kill the host cell. Therefore, TA-systems are hypothesized to stabilize mobile elements and to ensure that they are properly inherited after cell division [13, 41–44]. The stability of chromosomal integrons, which can contain more than 200 gene cassettes and often more than one TA-system [45], may thus be improved by these systems. In our gene catalog, we identified as many as 14 different classes of toxins and 15 classes of antitoxins of which 9 were part of the same system. This included, for example, BrnT/BrnA, RelE/RelB, ParE/ParD, HigB/HigA, YoeB/YefM and HicA/HicB. Several of these TA-systems have been previously found in integrons, where e.g. HigB/HigA have been detected in chromosomal integrons of *Vibrio spp*. [41] and HicA/HicB and HigA/HigB have been found in gene cassettes in humanassociated bacterial communities [46]. Another common gene found in the catalog was GST, which are detoxification enzymes that can catalyze glutathione to a wide range of xenobiotic substances [47]. Most common were glutathione-dependent formaldehyde-activating enzymes (Gfa), which condenses formaldehyde, a toxin commonly found in natural environments that binds and inactivates proteins [48, 49]. Previous reports have found GSTs and Gfa enzymes among integron-associated genes from marine sediments [35], and its prevalence suggests that this gene confers a selective advantage to the host cell.

Approximately two thirds (64%) of the genes in the catalog were ORFans, i.e. genes that did not match any known gene, function or domain in any entry in the reference databases. Genes with unknown function were also found to be highly overrepresented among the integron-associated genes. This suggests that a large fraction of the genes maintained in the integrons of the analyzed bacterial communities has uncharacterized biological functions or are too evolutionary distant to be annotated using sequence homology. These findings are in line with previous amplicon-based studies. For example, analysis of gene cassettes from deep-sea hydrothermal vents showed that up to 82% of their genes did not have any significant match in sequence databases [19]. Similarly, analyses of integrons in the human microbiome have demonstrated that up to 85% of the gene cassettes had ORFs of unknown function [23, 46]. The number of unknown genes present in environmental microbial communities has, regardless of their genomic contexts, shown to be substantial and can reach up to 80% [9, 35, 36, 50]. The exact role and biological functions of the large number of ORFans maintained by environmental bacteria is currently not clear. A recent study applied sensitive probabilistic alignment algorithms and showed that a proportion of the ORFans are likely to be distant homologs, which share too low similarity to known genes to be properly annotated using standard approaches [51]. Studies of the *Escherichia coli* pan-genome have, furthermore, shown that ORFans often have evolutionary conserved protein-coding capacity and that many are properly transcribed and translated [52, 53]. The large proportion of unknown genes in integrons found in our analysis further supports the hypothesis that many ORFans have important biological roles. Indeed, integrons are highly plastic and genes that would not provide any form of evolutionary advantage would be expected to be excised and not further maintained by the community. Thus, our results suggest that analysis of integrons using culture-independent techniques, such as shotgun metagenomics, may be used to guide the identification of novel mobile genes that encode previously uncharacterized biochemical functions that enables bacteria to adapt to different selection pressures [1, 54, 55].

Analysis of the gene catalog revealed that integron-associated genes were in general short with a length distribution centered around 400 nt. In contrast, chromosomal ORFs are often substantially longer, e.g. with a median of 849 bp in *Escherichia coli*. This difference was most likely not a result of the de novo identification of the ORFs, even if this sometimes estimates the length of the predicted gene incorrectly [27]. In fact, when we de novo predicted ORFs in the *Escherichia coli* genome using the exact same setting as in the analysis of the gene cassettes, the median length changed only slightly (from 849 to 831 bp). In addition, the lengths of the integron-associated genes predicted in this study were also relatively similar to the length of the genes reported in the integron database INTEGRALL (median length 474 bp). The short length of the ORFs is thus likely a biological effect and not a technical artifact, suggesting that the length of the genes present in integrons are under a strong evolutionary selection pressure. Indeed, it is well-known that incorporation of genes located on extrachromosomal DNA is typically associated with reduced fitness due to increasing the cost of cell replication, DNA maintenance and gene expression [56]. It can, however, not be excluded that there are other mechanisms associated with how genes are incised, excised and expressed in integrons that results in additional selection pressures on the gene length. Moreover, the G/C-content of the genes in the catalog varied substantially. Centered at an average G/C-content of 0.5, the distribution was more than twice as wide as the distribution encountered for chromosomal genes. The G/C-content of non-mobile chromosomal genes is known to differ significantly between bacterial genomes and has been shown to be correlated with genome size, habitat and living conditions, such as temperature [57–59]. Thus, the wide distribution of G/C-content among the integron-associated genes suggest that the ORFs have been mobilized from a diverse set of hosts with a wide range of different G/C-content. It also suggests that there is no, or little, selection pressures towards a more narrow G/C-content distribution.

Previous studies have demonstrated that integrons, in particular those of class 1, have gene cassettes that are dominated by antibiotic resistance genes [60–62]. This is especially true for clinical variants that are commonly occurring in pathogenic bacteria, such as Enterobacteriacea. In contrast, a recent systematic study of integron-associated genes in bacterial genomes demonstrated that only 4% of the genes could be associated with antibiotic resistance [6]. Our results, based on the analysis of DNA sequenced directly from bacterial communities, showed that only 0.19% of the genes were known antibiotic resistance genes. When relaxing the similarity threshold to also include genes that were homologous to known resistance genes, this number was still less than 0.5%. These findings show that integron-associated genes are, in general, not coding for antibiotic resistance determinants but typically contain genes with a much more diverse functional repertoire. It also suggests that integron-associated genes reported into existing repositories, such as INTEGRALL and GenBank, are likely to be heavily biased towards cultivable pathogenic bacteria and does only reflect a small proportion of the diversity present many environmental communities. Nevertheless, 38 resistance genes were present in our data. Interestingly, nine of these were novel and have previously not been reported in resistance gene databases. This suggests that integrons in environmental bacterial communities maintain resistance genes that have not yet been incorporated into human pathogens [24]. This is in line with previous studies that have reported a surprisingly large diversity of novel resistance genes present in metagenomes [63–66]. In fact, several forms of clinically relevant resistance genes have also been hypothesized to originate from environmental bacteria. In particular, *Shewanella spp.*, which are naturally occurring in marine ecosystems, have been suggested to be the source of resistance genes such as the beta-lactamase OXA-48 and the fluoroquinolone resistance gene qnrA [67, 68]. Another example are the beta-lactamases PER and MOX, which were recently shown to be mobilized from the bacterial genera *Pararheinheimera* and *Aeromonas*, respectively, which both are ubiquitously present in the environment [69, 70]. Thus, our results further underline that environmental bacterial communities are sources of antibiotic resistance genes. They also show that analysis of metagenomes can be used to identify novel integron-associated resistance gene variants that have not yet been encountered in clinical settings. It should, however, be pointed out that further experimental work is needed to confirm the phenotypes of the novel resistance gene variants.

The gene catalog presented in this papers was identified using a novel computational pipeline developed to search metagenomic data for integron-associated genes. The implementation can accurately identify *attC* sites directly, without requiring a complete integron to be present, which makes it applicable to the often short contigs produced by shotgun metagenomics. This is especially important when analyzing integrons, since they are often hard to assemble due to the presence of repetitive sequences [23, 25]. The computational pipeline combines two different steps to ensure a high performance. First, HattCI, a probabilistic model in the form of a generalized hidden Markov model was used to identify potential *attC* sites. The HattCI model uses pattern matching for the specific regions of the *attC* sites and therefore has both a high sensitivity and a high computational performance, which enables efficient processing of large data volumes. In the second step, HattCI predictions were evaluated by analyzing their secondary structure using a covariance model created using Infernal. Evaluation based on a 291 *attC* sites large testing set confirmed that this combination resulted in the ability to identify more than 90% of the *attC* sites. Furthermore, to keep the number of false positives to a minimum, we only considered *attC* sites that are found sufficiently close to each other. Since most integrons are expected to contain more than one gene, and thus more than one *attC* site, this efficiently removed many of the false positives caused by spurious isolated hits. Consequently, when analyzing 100,000 reshuffled *Escherichia coli* genomes, corresponding to ∼500 gigabases of sequence data, not a single false positive was found. However, the strict filtering suggest that there are likely additional integron-associated genes present in the analyzed datasets, e.g. singletons present on short contigs, that are not considered by our method. Furthermore, HattCI and the covariance model were trained using a gold standard dataset containing manually verified annotation of *attC* sites. The dataset is however based on data from public sequence repositories and therefore biased towards gene cassettes encountered in class 1 integrons [62]. Different classes of integrases are known to have different affinities for certain *attC* sites [71] and it is therefore likely that our approach misses many forms of *attC* sites. Consequently, the number of gene cassettes identified in this study is likely to be a conservative estimate. Reanalyzing the dataset when more comprehensive and unbiased training data becomes available is therefore supposed to further expand the presented gene catalog.

## Conclusions

In this study we present a systematic survey of gene cassettes present in metagenomic data. This is, to the best of our knowledge, the most comprehensive analysis of integron-associated genes in bacterial communities to date. We observed that the relative abundance of gene cassettes varies between communities, being more abundant in marine environments, and less common in the human gut. Also, our results show that integrons in bacterial communities maintain genes with a diverse set of biological functions. This is further emphasized by the large number of unknown genes for which no close homologue could be found in the sequence databases. Furthermore, the presence of previously undescribed antibiotic resistance gene variants supports the hypothesis that bacterial communities are a reservoir for novel resistance determinants that can be transferred into human pathogens. Our study also shows that metagenomic data can, regardless of its fragmented nature, be an important source of information for the characterization of integron-associated genes, and potentially other genes and genomic structures. Thus, further studies, including even larger volumes of metagenomic data, are warranted.

## Methods

### Description of the computational method

To accurately identify gene cassettes located on metagenomic contigs, a computational method was developed. The result, MIG-finder (Metagenomic Integron-associated Gene finder), operates by identifying the two main components of the gene cassettes: the recombination site (*attC*) and the associated open reading frame (ORF). First, the method predicts *attC* sites based on their sequence similarity using HattCI 1.0b which implements a seven-state generalized hidden Markov model (gHMM) to describe each conserved motif or variable region of the *attC* site (i.e. R’, R”, L’, L”, two spacers and loop). The gHMM was trained using 231 manually curated *attC* sites [15]. HattCI was run in the mode where both strands are analyzed, with a batch of 1000 sequences and 6 threads (*‘-b -s 1000 -t 6’*). All matches with a score above 0 was kept. Next, the secondary structure of the predicted *attC* sites was validated by a covariance model (CM) implemented using Infernal v1.1.1 [26]. The CM was constructed using the command cbuild in Infernal (using default parameters) from a structure-based multiple alignments produced by LocaRNA v1.8.9 [72] using 109 *attC* sites, which were chosen out of the initial 231 in order to produce a valid consensus for the *attC* site secondary structure. The model construction was guided by anchoring complementary positions in the alignment. Then, Infernal, in its maximum sensitive mode (*‘—max’*), was used to analyze all potential *attC* sites. *AttC* sites predicted with an Infernal score less than 20 were removed from the analysis. Due to the palindromic nature of the *attC* sites, overlapping matches could appear on both strands. In these cases, only the match with the highest score was selected. Next, the identified *attC* sites were filtered to remove false positives. This was done by only keeping *attC* sites located at a maximum distance of 4000 nucleotides from another *attC* site. This distance was assumed as the maximum distance of two *attC* sites located on the same integron. Consequently, *attC* sites found in isolation from any other *attC* site, i.e. more than 4000 nucleotides up or downstream were thus considered as potential false positives and discarded. Finally, ORFs were predicted using Prodigal v2.6.2 running in metagenomic mode [27], not allowing for genes to run off edges and saving the predictions in a fasta file with nucleotide and translated versions (‘-p meta -f gff -q –c’). Predicted ORFs were kept if they i) had a score larger than 0, ii) had maximum overlap of 50 nucleotides with any *attC* site and iii) for the first gene cassette in the array, the ORF was no further than 500 nucleotides away from the *attC* site. On the top strand, the predicted ORF was the associated with its closest upstream *attC* site, and reversely for the bottom strand. Note that, *attC* sites were required to be on the same strand to be part of the same integron.

The sensitivity of the method was evaluated using 291 manually curated *attC* sites [6] that represents the diversity of the *attC* sites present in INTEGRALL [16]. The pipeline was capable of detecting 90.4% of these sequences. The specificity was evaluated using data generated by reshuffling eight genomes, representing different part of the bacterial phylogeny, 10,000 times each (*Escherichia coli* (NC_000913.3), *Staphylococcus aureus* (NC_007795.1), *Bifidobacterium longum* (NC_004307.2), *Rhizobium marinum* (GCF_000705355.1), *Burkholderia cepacia* (GCF_001411495.1), *Acidobacterium capsulatum* (NC_012483.1), *Desulfobacter vibrioformis* (GCA_000745975.1), *Streptomyces griseus* (GCF_000010605.1)). For this data, the pipeline did not generate a single false positive.

### Processing of metagenomic data

The computational method was applied to 370 million metagenomic DNA sequences consisting either of assembled contigs or sufficiently long reads (average length of 712 nt). This data corresponded to approximately 10 Tbases of metagenomic sequenced reads. The data was compiled from four metagenomic databases, CAMERA [73], MG-RAST [74] [downloaded August 2016], NTenv [75] [downloaded March 2016] and EBI Metagenomics [76] [downloaded June 2016]. From the MG-RAST repository, only datasets that had at least 10,000 sequences and an average fragment length above 1,000 nucleotides were selected (i.e. containing contigs or long sequence reads). Redundant datasets that were present in several databases (e.g. CAMERA and MG-RAST) were removed. Ten additional metagenomic studies containing assembled contigs that were not present in the databases were also included [downloaded between Feb and July 2016]. One of these studies contained samples from marine periphyton biofilms from the coastal waters of the Swedish west coast collected and prepared by the authors. The other studies were identified through literature searchers and the data was downloaded from repositories or directly from the authors. In particular, Tara Oceans is a comprehensive study from epipelagic and mesopelagic communities around the world. For further details on the datasets see Table 1.

### Creation and analysis of the gene catalog

A catalog of integron-associated genes was created based on the ORFs and their corresponding *attC* sites predicted from metagenomic data. The catalog was made non-redundant by removing identical pairs of *attC* sites and ORFs (100% sequence similarity for both, in their nucleotide and aminoacid sequences, respectively). Each ORF in the catalog was functionally annotated using three independent general databases containing gene functions: Cluster of Orthologous Groups (2003-2014 COG) [28], TIGRFAM 15.0 [29] and PFAM 29.0 [30]. Each ORF was compared against each database using hmmscan in HMMER 3.1b [77] with default parameters and saving a table of hits per-sequence. Only the best match per sequence, with a maximum E-value of 0.001 was kept. Gene Ontology analysis was done by mapping the PFAM hits to the metagenomics GO-slim annotation [78]. Next, the gene catalog was compared with three specialized databases: the INTEGRALL [16], ResFinder [31] and BacMet [32]. The comparison was performed using BLAST v2.2.31+ [79] in ‘tblastn’ mode for INTEGRALL and Resfinder and in ‘blastp’ mode for BacMet. The comparison was done using two separate cut-offs, one strict (97% sequence similarity) and one relaxed (70%). In both cases, only matches that covered at least 50% of the subject were kept. If there were multiple matches, the match with the highest sequence similarity in each database was kept.

All unique *attC* sites were clustered based on sequence-structure similarity using GraphClust [33] with default parameters modified to “input_win_shift = 100, input_win_size =200, OPTS_fasta2shrep_gspan = ‘-t “3=0,5=80” -M 5 -c 20 --cue -u --stack --seq-graph-t –seq-graph-alph’, GLOBAL_iterations = 4 and GLOBAL_num_clusters = 2”, which pre-defined the number of clusters to be 8. The resulting cluster were manually evaluated to contain the key *attC* site motifs, i.e. R”/R’ and matching L”/L’, with an extra-helix nucleotide formed in L” due to difference in length with L’, and in addition the presence of second extra-helix nucleotide found in the loop [71]. Then clusters with a structural consensus that did not contained these features were discarded, resulting in 5 structural clusters. Tests for functional enrichment of COG Categories and Gene Ontology were done for each generated cluster. The test was done using Fisher’s exact test [80] comparing the relative number of occurrences of a specific function within the cluster compared to all other ORFs in the catalog. For the test, ORF’s that did not have a functional annotation were discarded together with their *attC* site. Similarly, *attC* sites that did not belong to one of the five valid structural clusters were discarded together with their ORFs.

## Supplementary information

**Additional file 1Table S1**. The full catalog of integron-associated genes identified in this study. Details for all attC sites and integron-associated genes presented in cassette structure, including sequence and functional annotation. Note that in order to preserve the cassette structure, genes and attC sites redundancy has not been removed.

**Additional file 2Figure S1**. Functional annotation of the integron-associated genes using TIGRFAM functional roles. Of the 13,397 integron-associated genes in our catalog 1203 genes matched a TIGRFAM with a known function. Percentages on the plot are given in relation to those numbers

**Additional file 3Table S2**. Gene ontology analysis of the integron-associated genes using PFAM families [30]. Out of the 13,397 non-redundant integron-associated genes in our catalog, 3488 matched a PFAM family with a known function, which were in turn mapped to the metagenomics GO slim [78]. Note that the GO terms are organized in decreasing order inside each category, molecular function, biological process and cellular component.

**Additional file 4Table S3**. Overrepresentation analysis of each COGs category between integron-associated and non-integron-associated genes found in the metagenomes. For each category, overrepresentation is given as odds ratio and the p-value of Fisher exact test.

**Additional file 5** Secondary structural consensus for the 5 distinct clusters of *attC* sites. The clusters were generated by GraphClust.

**Additional file 6Table S4**. Over-representation test using Fisher’s exact test for COG functional categories [28]. Significant *p*-values are shown in bold.

**Additional file 7Table S5**. Over-representation test using Fisher?s exact test for GO-terms found in the metagenomics GO slim [78]. Significant p-values are shown in bold.

## Data Availability

The data analyzed in this study consisted of pre-existing datasets which are specified in Table 1.

## Abbreviations

CM: Covariance model
COG: Cluster of orthologous groups
gHMM: Generalized hidden Markov model
ORF: Open reading frame
PCR: Polymerase chain reaction
