# Supplementary figures and images for: A comprehensive survey of integron-associated genes present in metagenomes
Source: BMC Genomics. 2020 Jul 20;21:495. doi: 10.1186/s12864-020-06830-5 (PMC7370490; doi:10.1186/s12864-020-06830-5)

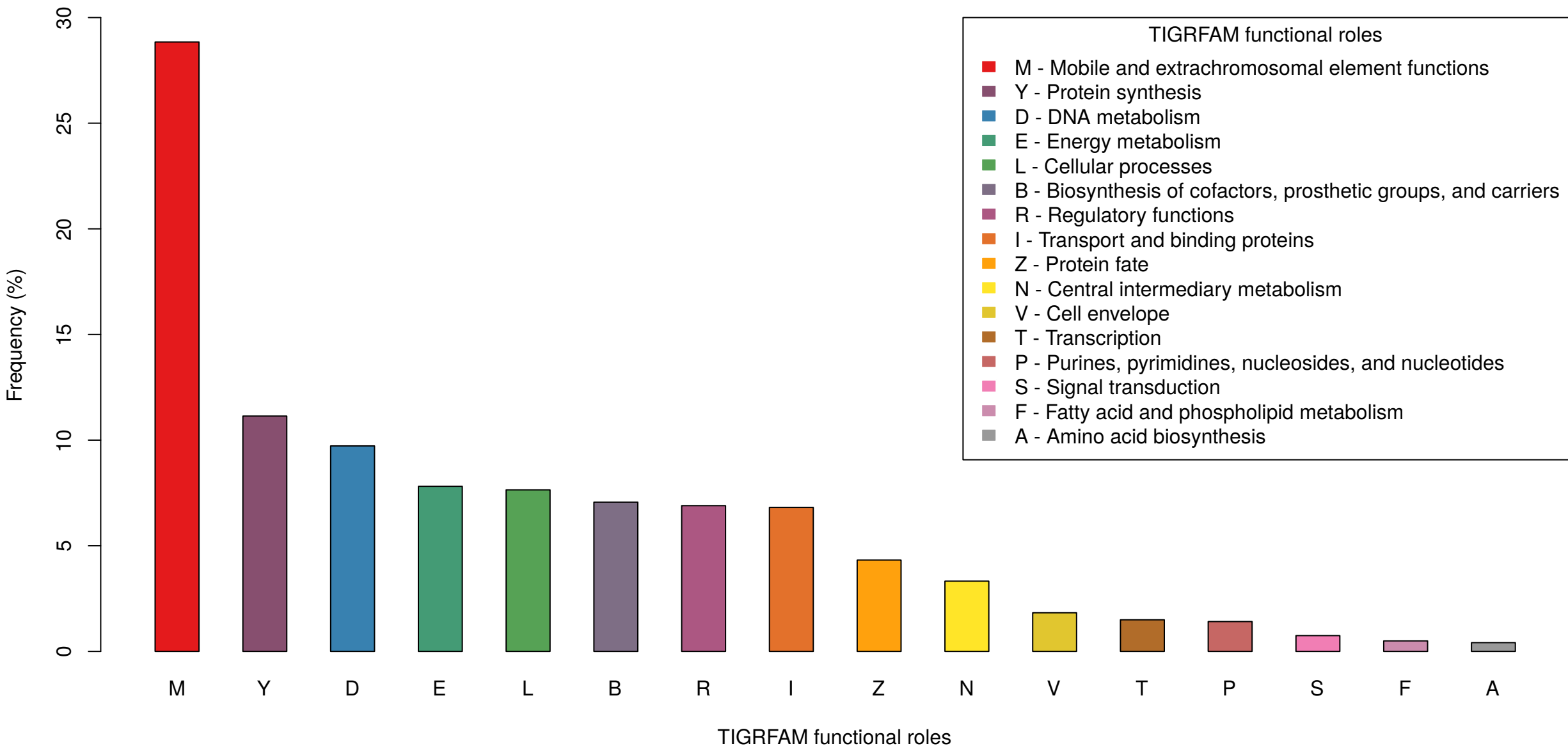

Supplement: Supplementary file 2 — Additional file 2Figure S1. Functional annotation of the integron-associated genes using TIGRFAM functional roles. Of the 13,397 integron-associated genes in our catalog 1203 genes matched a TIGRFAM with a known function. Percentages on the plot are given in relation to those numbers [file 12864_2020_6830_MOESM2_ESM.pdf]

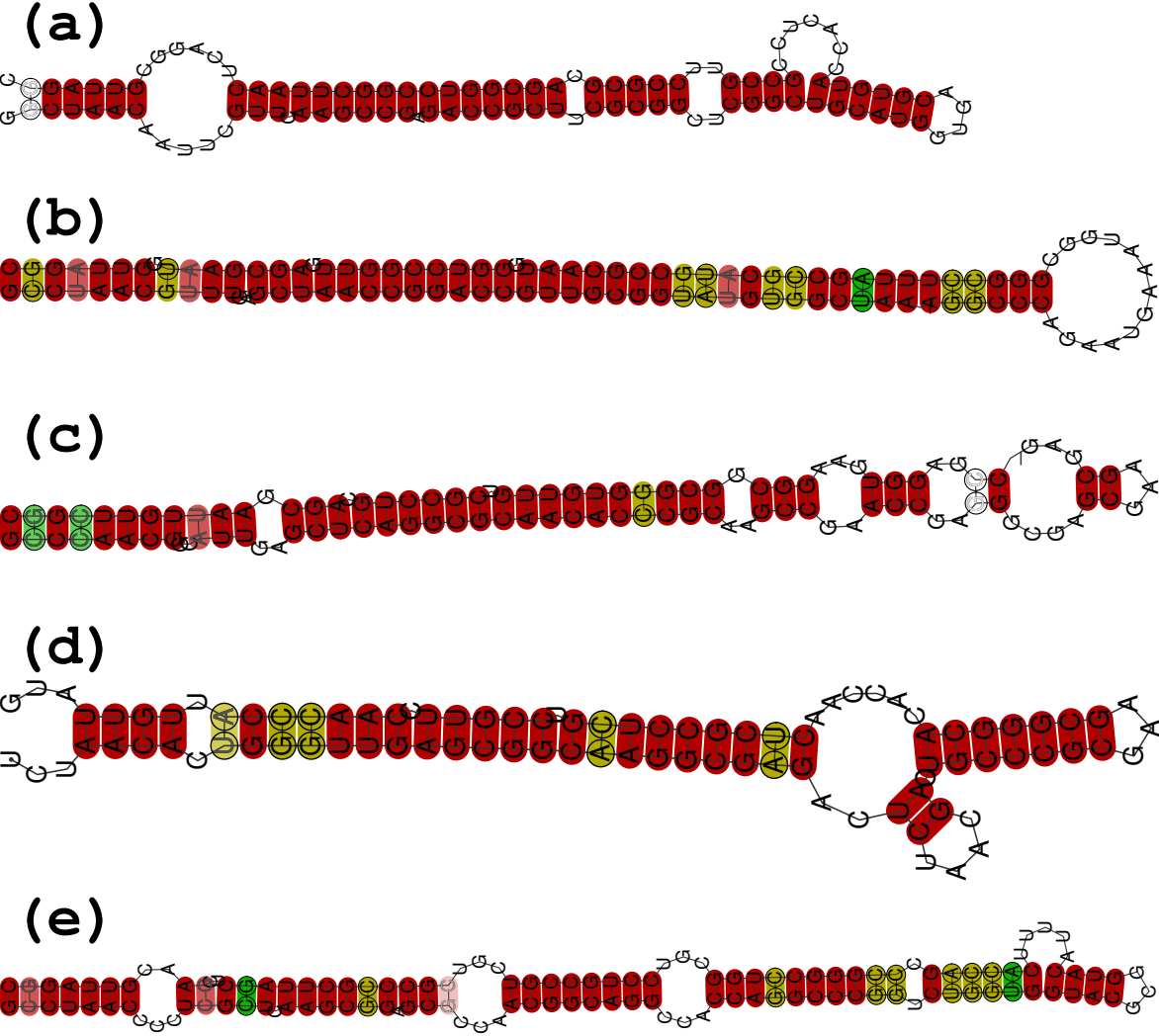

Supplement: Supplementary file 5 — Additional file 5 Secondary structural consensus for the 5 distinct clusters of attC sites. The clusters were generated by GraphClust. [file 12864_2020_6830_MOESM5_ESM.pdf]
